# Assessing excellence in translational cancer research: a consensus based framework

**DOI:** 10.1186/1479-5876-11-274

**Published:** 2013-10-29

**Authors:** Abinaya Rajan, Carlos Caldas, Henri van Luenen, Mahasti Saghatchian, Wim H van Harten

**Affiliations:** 1 The Netherlands Cancer Institute, Amsterdam 1066 CX, The Netherlands; 2 Cancer Research UK, University of Cambridge, Cambridge CB2 0RE, UK; 3 Institut Gustave Roussy, Villejuif 94805, France

**Keywords:** Translational research, Cancer, Excellence, Assessment, Criteria, Framework, Stakeholder consensus, Peer-review

## Abstract

**Background:**

It takes several years on average to translate basic research findings into clinical research and eventually deliver patient benefits. An expert-based excellence assessment can help improve this process by: identifying high performing Comprehensive Cancer Centres; best practices in translational cancer research; improving the quality and efficiency of the translational cancer research process. This can help build networks of excellent Centres by aiding focused partnerships. In this paper we report on a consensus building exercise that was undertaken to construct an excellence assessment framework for translational cancer research in Europe.

**Methods:**

We used mixed methods to reach consensus: a systematic review of existing translational research models critically appraised for suitability in performance assessment of Cancer Centres; a survey among European stakeholders (researchers, clinicians, patient representatives and managers) to score a list of potential excellence criteria, a focus group with selected representatives of survey participants to review and rescore the excellence criteria; an expert group meeting to refine the list; an open validation round with stakeholders and a critical review of the emerging framework by an independent body: a committee formed by the European Academy of Cancer Sciences.

**Results:**

The resulting excellence assessment framework has 18 criteria categorized in 6 themes. Each criterion has a number of questions/sub-criteria. Stakeholders favoured using qualitative excellence criteria to evaluate the translational research “process” rather than quantitative criteria or judging only the outputs. Examples of criteria include checking if the Centre has mechanisms that can be rated as excellent for: involvement of basic researchers and clinicians in translational research (quality of supervision and incentives provided to clinicians to do a PhD in translational research) and well designed clinical trials based on ground-breaking concepts (innovative patient stratification, substantial fraction of phase I/II trials, investigator-initiated trials). Critically, the framework supports reduced bureaucracy by building on existing European evaluation systems.

**Conclusions:**

The excellence framework is the product of an intense stakeholder consensus building exercise. It will be piloted during an expert peer review/site visit of at least three European Comprehensive Cancer Centres. The findings regarding content, governance and implementation can have relevance for other clinical and research fields.

## Background

Translational research can be defined as a complex process of transforming scientific discoveries, arising from laboratory, early clinical, or population studies, into clinical applications to reduce incidence, morbidity, and mortality [1]. On average, it takes over a decade to deliver patient benefits [2,3]. After the 2007/09 financial crisis healthcare providers find it harder to justify funding for translational research in cancer but also other fields. The Stockholm Declaration recognises that creating a strong case for funding translational research in Europe, needs proof of excellent performance by Cancer Centres that are engaged in it [4]. Assessing excellence can stimulate continuous improvement in the way an organization perceives, plans, and performs translational research for the benefit of patients. It can also help to develop a network of excellent Centres that can focus their collaboration and share best practices through regular benchmarking.

“*In the last decade*, *we have begun discussing the idea of translational research in every field of medicine without ever clarifying exactly what this entails and how it should be assessed.*” (*Researcher*)

To date, no assessment framework has specifically focused on excellence along the entire continuum of translational research. Previous frameworks have focused on criteria/questions mainly for the self-guided assessment of the success of translational research organizations and/or projects [5,6]. But there are a number of limitations to those frameworks: (i) not every success may necessarily be a sign of excellence; (ii) self-assessment is not sufficient to benchmark the performance of different Centres. Although success in translational research may be assessed by the organization itself, for the sake of credibility, excellence assessment requires an expert judgement (preferably at an international level) that is completely independent to the organization. Currently, no formal framework exists that supports peer-reviewers to judge excellence in translational research; (iii) previous frameworks were informed by a few experts but did not engage key stakeholders in setting criteria. Achieving excellence in translational research relies on people from different disciplines and functions working together to improve overall performance. Consensus building helps achieve common understanding, commitment and collaboration and is recommended for criteria development [7].

This study produced an excellence assessment framework that was developed using a consensus building exercise with key stakeholder groups and experts from the European cancer community. The framework will be used to identify and assess excellent translational research in a number of European Comprehensive Cancer Centres (CCC’s) (combining basic, translational and clinical research and patient care activities). The framework will be thoroughly piloted with a number of CCC’s in 2013-2014.

## Methods

**Figure 1:**
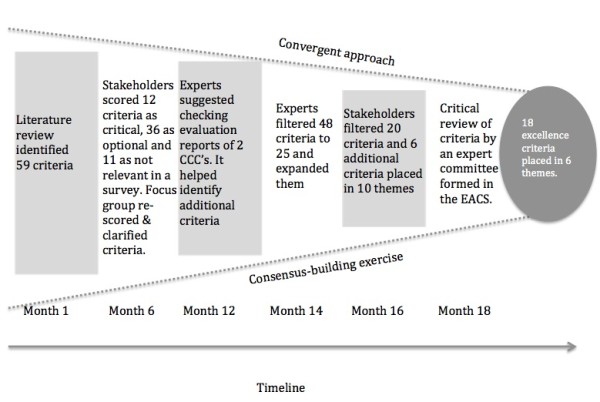
Consensus building exercise with stakeholders to derive an excellence framework for assessing translational cancer research.

Consensus building took 18 months using several methods to fully engage stakeholder groups (See Figure 1). These included clinicians, researchers, senior managers and patient representatives, representing around 70 European organizations including: CCC’s, Cancer Research Centres, Clinical Cancer Centres, Cancer Units, Patient Organizations and Cancer networks. Recruiting a small group of seven acknowledged experts to provide informed review and reflection at key points complemented this process. Development of the excellence assessment framework can be summarized as follows:

•*Literature reviews* – The European accreditation standards for CCC’s from the Organization of European Cancer Institutes (OECI) and a report from the National Cancer Institute (USA) Translational Research Working Group (TRWG) on improving translational research performance [8] were taken as a starting point. A systematic literature review followed to identify and critically appraise translational research models most suitable for performance assessment of CCC’s [9]. The result was a list of 59 excellence criteria covering inputs, processes and outputs of basic, translational and clinical research & clinical care.

•*Stakeholder survey and focus group discussion* – This initial list of 59 criteria was scored by stakeholders (N = 78) in an online survey. Evaluation of criteria by participants identified criteria as critical (if selected by more than 60% of the participants as important), optional (if selected by 40-60% of the participants as important) or not relevant (if selected by less than 40% of the participants as important). 12 of the 59 criteria scored critical (*e*.*g*. *early stage clinical trials*; *effective transfer of innovations from basic research to clinical practice*); 36 optional (*e*.*g*. *improved Quality of Life from innovations implemented*; *innovative prevention services*) and 11 not relevant (*e*.*g*. *number of surgical*/*paediatric*/*radiotherapeutical subspecialities*; *number of radiotherapy units*). Quantitative criteria generally scored lower than qualitative criteria. Next, a focus group was assembled with a representative sample of survey participants (N = 30). Paricipants clarified that the criteria that scored critical and optional should be considered when developing excellence criteria and the criteria that scored not relevant should be discarded with the exception of Health Technology Assessment.

•*Expert Group meeting* – The updated list was sent to an expert group (2 basic, 2 translational, 2 clinical experts and 1 senior management expert). Their selection reflected more than 30 years of experience in basic, translational and/or clinical cancer research, prestigious awards and memberships in Oncology, significant current roles in European Comprehensive Cancer Centres and willingness to contribute to excellence framework development. The experts suggested a comparative review of the adapted list against recent external peer-reviewed evaluation reports from two CCC’s (see acknowledgements) to check if the criteria were reflected in these reports and to what extent additional criteria can be identified. The revised list was sent back to the experts who scrutinized each criterion, added specific points, and placed them under 10 categories that shaped an excellence assessment framework. The expert group stressed the importance of using expert peer-review when assessing the quality of translational research in combination with the assessment framework being developed.

•*Final validation by stakeholders* – The revised version of the assessment framework was sent to the same stakeholders who had previously participated in the consensus building exercise. The participants (N = 34) made suggestions to improve criteria clarity in terms of being to the point, having short sentences, avoiding connectors such as “and”/”or” to make them less risky for misinterpretation. Ultimately, stakeholder feedback helped filter and refine the list down to 20 excellence criteria to assess excellence in translational research in CCC’s during a peer-review process and 6 additional criteria to be considered according to the preference of the CCC’s.

•*Critical review of excellence criteria by an external committee within an independent body*, *the European Academy of Cancer Sciences* (*EACS*) - The excellence criteria that evolved from the stakeholder consensus building were critically reviewed by a committee (see endnote for the composition^a^) that has been formed in the EACS to give external input and govern the excellence assessment. The committee suggested minor re-structuring to the excellence criteria. They reduced the criteria to 18 core excellence criteria, placed them in 6 themes and merged all additional criteria with the core criteria (see Table 1).

**Table 1:** Excellence framework for assessing translational cancer research

**Excellence criteria**	**Sub-criteria/questions to help peer-reviewers assess cancer centres**
**Theme 1. Organizational policies and strategies**	
Evidence for integration of Basic, Translational, and Clinical research with excellence in all areas	Effective communication between multidisciplinary teams?
Centre is treating patients in at least 3 major cancer types at an internationally competitive level	Sufficient patient volume?
	Appropriate infrastructure?
	Internationally recognized medical specialists?
	Expertise level?
Mechanisms are in place for continuous quality assurance.	Defined protocols for:
	Output monitoring?
	Peer review programs?
	Ethical standards?
	Teaching good practices?
	Scientific misconduct provisions?
**Theme 2. People management**	
Clear recruiting strategy to promote excellence	Internationally competitive recruiting?
	Attention for gender issues?
Independence of PIs is clearly defined	Defined institutional support for PIs?
	Incentives to improve leadership competencies in place?
The research program of PIs is regularly evaluated	Scientific output?
	Multidisciplinary activities?
	Regular site visits?
Mechanisms are in place to involve basic researchers and clinicians in translational research	Active participation of clinicians in basic/translational research?
	Institute clearly facilitates participation?
	Is interaction between clinicians and basic researcheffectively stimulated?
	Number of clinicians participating in MD-PhD programs during last 5 years?
Mechanisms to promote collaboration with research teams outside the Centre	Number and quality of joint output?
	Partners are internationally leading?
**Theme 3. Research infrastructure/competencies**	
Centre has internationally competitive facilities and proven forefront expertise in a substantial number of key areas.	Prominence in number of the following areas:
	- Identifying, validating, and designing rational Rx strategies directed at key molecular cancer targets?
	- Surgery, innovative operation theaters.
	- Radiotherapy infrastructure?
	- Next generation sequencing and other “omics”?
	- Bioinformatics and computational biology (both infrastructure and innovation)
	- Robotic screening (drugs, shRNA, siRNA)?
	- Advanced microscopy facilities (e.g. confocal, lifetime imaging, flow cytometry etc)?
	- Clinical imaging and innovative modalities?
	- Prominence in area of animal model systems?
	- State of the art biobank with clinical informatics linked with genomic and other data?
	- Patient registry with strong biostatistical support?
	- PK, PD monitoring phase 1/2 clinical trials?
	- Pharmaceutical production/formulation?
	- Production biologicals for use in patients?
	- Molecular pathology?
	- Good interface with chemistry, physics, engineering, mathematics etc?
	- Population studies and resources such as cohorts?
	- Health economics; primary care links; early detection programmes?
	- Technology Transfer support?
	- Other?
**Theme 4. Clinical (trial) management**	
Clinical trials are well designed	Number of innovative aspects:
	- Has it performed groundbreaking proof of Concept trials? Were these based on molecular tumor parameters?
	- Innovative stratification of patients (adaptive trial design)?
	- Investigator-initiated trials?
	- First in man?
	- Substantial fraction of phaseI/II trials?
	- Advanced modeling (e.g. PDX)?
Centre utilizes an internal review system to select for the most innovative and promising protocols.	Evidence that this has lead to innovative trials over a 5-year period?
Patients enrolled in clinical trials	A substantial fraction (>10%) of patients is enrolled in phase I/II trials?
Continuous improvement of the quality of patient care	Appropriate monitoring with patient participation in the process?
Outcome is at forefront and based on patient mix treated	Proper benchmarking?
**Theme 5. Internationally recognized excellence**	
Research has resulted in changes in clinical thinking and practice – emphasis on physician investigators.	- Examples to be listed.
	- Best in class young and mid career physician-investigator faculty recruited and retained by the Centre
	Is the Centre training and recruiting ever better physician/oncologist-investigators?
The Centre has an international reputation ranking it in the top 10% segment	Evident from:
	- Output related to size and expenditure based on independent benchmarking performed within last 3 years.
	- Substantial impact is evident in all three research areas (basic, translational, clinical).
	- High rating by international peers
	- Prestigious collaborations
	- Accreditation status
	National/international awards
	- Prestigious competitive grants obtained
**Theme 6. Financial expertise**	
Efficient financial management and support	Appropriate support for managing external grants and clinical research projects including contracts with industry.
A substantial fraction of income is obtained through funding bodies that employ a critical review process.	Objective success in open competition for grants.

## Results

The consensus building exercise clearly identifed a need to assess excellence in translational research based on qualitative rather than quantitative criteria. Stakeholders and experts felt that whilst for instance state-of-the-art infrastructure is important to perform excellent translational research, the assessment of excellence itself should focus more on how efficiently they are being used by the organization and the quality of their outputs.

“*The numbers of services*/*units or treatments required to be assessed as* “*Excellent*” *appear to me to be somewhat arbitrary*. *Quality rather than quantity should prevail*.” (Researcher)

There was unanimous support for the need to minimize the bureaucracy of the excellence assessment and to have an external expert-based governance system that is independent to the organization being assessed in order to maximize transparency. The experts suggested that reports regularly prepared by the CCC’s for existing national and/or European level assessment programmes should be first assessed against the excellence criteria (Table 1). These reports contain sufficient qualitative and especially quantitative data and only data missing in such reports needs collecting. A European excellence assessment framework can only be established if the procedure exceeds the current national accreditation efforts within the European Union (EU) member states and carries sufficient credibility.

“*CCC*’*s in the European Union member states already go through different expert based peer*-*reviewed national evaluations*, *how are we going to justify the need for and reduce the bureaucracy of a European excellence assessment*?” (*Experts*)

It was emphasised that assessing the translational research “process” along the entire continuum from basic research upto clinical practice and back is as critical as assessing inputs and especially outputs. However, it was decided that the assessment does not have to encompass population based outcomes because that stage goes beyond the scope of most European CCC’s and is the responsibility of different authorities within the EU member states. Furthermore, it has a very long lagtime and is influenced by several non-institutional factors which makes it difficult to access data on time. A focus on organizational level assessment was preferred. However, for certain issues, such as those related to prevention and early detection, translational research should extend its scope beyond current organizational boundaries to optimize its relevance on population level. Stakeholders can be made aware of this through relevant contacts with Europe-wide organizations such as the European Public Health Alliance (EPHA) [10] who act as gatekeepers to the national and regional authorities across the EU-28 member states.

“*It is important to establish an excellence assessment*, *which helps assess the highest levels of translational research quality through the innovation*, *productivity and efficiency of an organization.*” (*Manager*)

Another example of careful qualitative assessment instead of quantitative data collection is the use of the scientific infrastructure that CCC’s offer to other institutions. Such criteria cannot be imposed on all CCC’s as the nature of demand depends on specific regional/national contexts.

“*Of course*, *an excellent Centre should be willing to make these facilities available if asked for on a collaborative basis*. *But at my Centre*, *while we make some of these facilities available across the rest of city*, *we have not in the past five years had any requests to make these facilities available to other Centres in the nation because these Centres have their own access to these facilities.*” (*Manager*)

Finally, excellence criteria on patient-related aspects were perceived to be specifically important.

“*We must not only look at efficacy and safety in clinical study setting but even more to the value in the everyday practice of products*/*technologies*/*therapies.*” (*Patient representative*)

## Discussion

By undertaking a consensus-building exercise to develop an excellence assessment framework it was clear that while consensus can exist at a general level, some disagreement is unavoidable due to the different backgrounds, experiences and interests of the stakeholder groups. For instance, clinicians and patient representatives felt that excellence assessment does not need to involve basic research and should focus more on clinical care, but basic researchers and managers felt that unless basic research is included, the entire continuum of translational research cannot be fully assessed. Furthermore, the question whether the right stakeholders and experts are involved was carefully addressed at each stage of the criteria development process. For each excellence criterion, we considered having consensus when 60% or more of the stakeholders that participated agreed and we did not consider single votes by an individual stakeholder as sufficient. Inadvertently, some perspectives may have been left out that might still show to be critical. Excellence assessment requires a degree of flexibility, which is possible to implement in a transparent manner by using an independent peer review panel. While a common format is desirable, rigid formats may be unsuitable for organizations operating in different health systems and can introduce significant bureaucracy.

Qualitative excellence criteria increase the challenge for objective rating. A flexible and meaningful rating system is therefore needed. For the final decision, strengths and weaknesses across all criteria as well as individual criterion should be considered with an agreed minimum score on each criterion. However, to what extent should there be flexibility in accommodating the limitations imposed on individual CCC’s because they have to operate in different European health systems? Regarding health care, the EU operates on a subsidiarity principle. It means that all clinical and some research fields operate within nationally set frameworks. However, transnational cooperation is valued to share knowledge and improve performance and this brings some specific rules into play. Most EU member states have competitive national assessments but none would be easily accepted for use in another member state. Instead, considering best practices from across all member states in identifying and assessing excellence in translational research is far more transparent and can give wider acceptance of such assessments across the EU. Essentially, it is about an individual Centre making its own case of why it deserves to be designated as excellent given its own operating enviornment. And then the peer-reviewers can check whether the case made by the Centre is valid during a site visit. This will be done with the help of the excellence criteria (Table 1) to some extent but experts should also be prepared to come across areas of excellence that the Centre may have forgotten to mention during its application for excellence designation. A related issue raised by some experts was about discarding or “killing” insufficiently promising translational research projects, because of insufficient innovation, a low chance of clinical implementation or probably very unfavorable cost effectiveness. Of course identifying those is a risky matter, as it is often very difficult to predict the actual clinical potential in early stages of research. Nevertheless and increasingly, early stage Health Technology Assessment techniques are being developed and applied to aid decision makers to decide about further research investments or researchers to set the specifics/demands that the research and development process should meet [11]. One could consider looking into the availability of mechanisms to assist early stage decision-making on adequate translational research progress. It seems advisable to develop specific knowledge and development of a norm or reference material on this topic.

“*Research is also a matter of intuition*, *intellectual flexibility and aptitude to identify opportunities*. *Excellence assessment must go beyond putting a tick against criteria and needs to consider the local context in which institutions operate.*” (*Manager*)

For example, for high impact of publications it was hard to establish a minimum level because it varies greatly between the different disciplines within Oncology and among CCC’s across the EU member states. However, the average scores for each discipline can still be considered to make this criterion inclusive. Further, a range of bibliometric index other than just impact factors should be considerd i.e. citation factor, cumulative impact factor and the quality and impact of individual publications to be rigorously judged by an expert peer review team.

“*One publication that shows the 100*% *cure of a cancer is enough*. *Ten publications of one*-*month prolongation of survival mean nothing.*” (*Clinician*)

In Europe, the current excellence assessment primarily intends to evaluate team science due to the multifaceted nature of translational research where collaboration of different disciplines is critical to its success. The criteria that have emerged through the consensus building exercise, support this statement for example by focusing on multidisciplinary team collaboration, communication and joint publication efforts, participation of different department staff in various research projects and the outputs. A specific product of work may involve biologists, medical chemists, pharmacologists, imaging physicists and clinicians. Thus it can be difficult to identify the exact contributions made by each single member of the team and this could raise issues when individuals are evaluated for tenure or promotions etc. However, the consensus building exercise revealed that also monitoring individual efforts to some extent is needed for excellence. This could help promote a competitive attitude among researchers within and outside a Centre and help identify and reward excellent contributions of specific researchers to science that might otherwise go undetected. The individual efforts will be evaluated taking into account a range of factors: the quality of scientific outputs by clinicians pursuing a PhD in translational research; the quality of the research programs of PI’s and if they are regularly evaluated; prestigious awards, discoveries and memberships attributed to specific individuals from the Centre; investigator-initiated trials and their success rate etc. Currently, national assessment programs within some EU member states conduct these evaluations but there is no formally agreed assessment on European level. Finally, this consensus building exercise revealed a need to get some obvious basics right that might otherwise be ignored. For example, to ensure that the criteria are both meaningful and easily understood by organizations, words such as “high”, “well”, “minimum”, “significant”, “cutting-edge”, “state-of-the-art”, “substantial” etc. unless carefully explained can be easily misinterpreted by stakeholders. Stakeholders accepted to start developing an excellence framework with these words/definitions but suggested refining them based on pilots.

### Piloting of the excellence framework

A committee consisting of internationally respected and renowned experts in Oncology will govern the excellence assessment process. In the EU, members of the European Academy of Cancer Sciences satisfy such requirements, and international experts (from the National Cancer Institute, USA and Accreditation Canada) will be invited to join the committee and the official peer review team. A Centre that applies to be assessed as excellent should provide documentation to the committee. This will include recent external peer reviewed evaluation reports in English that the Centre has produced for national and/or international evaluations in the past 3 years, covering basic, translational and clinical areas with specific achievements in translating innovations from bench to bedside and/or vice versa. Further documentation may be requested if the initial material is found insufficient. After an initial screening of the documents against excellence criteria (Table 1) the committee will decide if the Centre qualifies for site visit/peer review in which again the excellence criteria will be used to evaluate Centres. A minimum of three European CCC’s will pilot the excellence framework.

## Conclusions

Assessing excellence requires a mix of quantitative and qualitative criteria retrievable through different data sources. But we need to recognise that the consensus building exercise showed strong support for qualitative criteria. This is because it will build on existing evaluation systems across the EU and other international systems (e.g. US, Canada) that already provide the necessary breadth of quantitative data. The assessment framework that we have developed will need to be thoroughly tested with European CCC’s to prove that it can help identify excellence in translational research. Although, the framework was primarily developed for Oncology, it can probably be translated to other research and/or clinicial fields after rigorous validation. Allocating governance to an external entity that has credibility and is independent of the organizations being assessed is a key ingredient. Finally, the success of the assessments will depend on minimized bureaucracy and maximized transparency and accountability during the evaluation process.

## Endnote

^a^The composition of the committee formed in the European Academy of Cancer Sciences is: Prof. Dr. Anton Berns (Senior Group Leader Molecular Genetics, Netherlands Cancer Institute), Prof. Dr. David M. Livingston (Chairman, Executive Committee for Research, Dana-Farber Cancer Institute and Emil Frei Professor of Genetics and Medicine, Harvard Medical School), Prof. Dr. Daniel Louvard (Director of Research Centre at Institut Curie France), and Prof. Sir Bruce Ponder (Head of Department, Oncology at University of Cambridge, UK).

## Competing interest

The authors declare that they have no competing interests.

## Authors’ contributions

AR and WvH conceptualized and designed the consensus building exercise. AR collected and assembled the data including inviting staleholders and experts to contribute to the development of the excellence framework. AR, HvL, CC, MS and WvH all gave input to analysing and interpreting the data. AR and WvH wrote the draft manuscript which was significantly improved due to the suggestions and comments made by all other authors HvL, CC and MS. All authors read and approved the final manuscript.

## Authors’ information

AR is a PhD Candidate at the Netherlands Cancer Institute, Amsterdam/University of Twente on a European project Eurocan Platform that is funded by the European Commission. Her thesis focuses on identifying and assessing excellent performance in translational research at the Comprehensive Cancer Centres in the EU. Prior to this, she holds a Masters degree in European Regional Health systems including expertise in health policy, patient advocacy, social science and Health Technology Assessment. CC is a Professor at the Cambridge Research Institute, UK who leads his own research group on Breast Cancer Functional Genomics using genomics tools such as sequencing, molecular cytogenetics, array-CGH, mRNA/miRNA profiling etc. MS is a Medical Oncologist at Breast Cancer Unit of the Institut Gustave Roussy, Villejuif, France where she is also responsible for European and international affairs. She is the chairwoman of the Accreditation and Designation programme of the OECI. HvL is a post-doctoral researcher in basic cancer research. He is currently also the manager of research operations at the Netherlands Cancer Institute, Amsterdam. WvH is a member of the board of Directors of the Netherlands Cancer Institute, Amsterdam. He is an Associate Professor at the Department of Health Technology and Services Research at the University of Twente, The Netherlands. He is also the president of the OECI.
